# Knowledge, attitudes, and practices regarding dogs and dog bites in Indigenous northern communities: A mixed methods study

**DOI:** 10.3389/fvets.2023.1080152

**Published:** 2023-02-20

**Authors:** Laurence Daigle, André Ravel, Yves Rondenay, Audrey Simon, Kabimbetas Noah Mokoush, Cécile Aenishaenslin

**Affiliations:** ^1^Département de pathologie et microbiologie, Faculté de médecine vétérinaire, Université de Montréal, Saint-Hyacinthe, QC, Canada; ^2^Groupe de recherche en épidémiologie des zoonoses et santé publique (GREZOSP), Faculté de médecine vétérinaire, Université de Montréal, Saint-Hyacinthe, QC, Canada; ^3^Centre de recherche en santé publique de l'Université de Montréal et du Centre intégré universitaire de santé et de services sociaux (CIUSSS) du Centre-Sud-de-l'île-de-Montréal, Montréal, QC, Canada; ^4^Centre Hospitalier Universitaire Vétérinaire, Faculté de médecine vétérinaire, Université de Montréal, Saint-Hyacinthe, QC, Canada; ^5^Independent Researcher, Kawawachikamach, QC, Canada

**Keywords:** dog bite, epidemiology (EPI), Indigenous, northern community, public health, rabies

## Abstract

**Introduction:**

The singular relationship developed over the years between northern Indigenous peoples and dogs has been profoundly changed through historical trauma, settlements and increased use of snowmobiles. Issues related to dogs have become increasingly complex and worrisome with the endemic presence of the rabies virus among Arctic fox populations, and given the fact that northern Indigenous peoples may have a higher risk of dog bites than the general population. This study aimed to investigate factors related to the risk of dog bites in Naskapi and Innu communities located in northern Quebec (Canada) by (1) describing the knowledge, attitudes and practices (KAP) regarding dogs and dog bites in these communities, and (2) analyzing experiences of inhabitants and health professionals with regard to dog bites and their management.

**Methods:**

A mixed methods study design that combined an observational cross-sectional survey and individual interviews was used. The survey collected data on KAP regarding dogs and dog bites among 122 respondents. Individual interviews (*n* = 37) were then conducted with victims of dog bites, owners of dogs that have bitten a person before, and health professionals. Descriptive and inferential analysis (quantitative data) and thematic analysis (qualitative data) were performed.

**Results and discussion:**

Results highlighted that 21% of respondents have had a dog bite in their lifetime. Most respondents were not aware of the risk of contracting rabies following a dog bite, although rabies risk perception was associated with risk perception of dogs (linear regression: coefficient = 0.69, 95% CI = 0.36–1.02). The odds of being more knowledgeable on rabies were higher (logistic regression: OR = 2.92, 95% CI = 1.07–7.98) among young adults. Dogs were perceived as both threats and protectors by community members. When the fear of dogs was present, it affected the quality of life of some inhabitants. There was confusion about responsibilities in the management of biting dogs, although protocols to follow after a bite were clear for health care professionals. This study revealed a lack of awareness and knowledge about dog bites and rabies risks in both communities. Results provide important knowledge for the development of interventions adapted to northern Indigenous communities.

## 1. Introduction

Dog bites have been studied in many contexts over the past decades due to their health impacts. Bites can cause short- or long-term physical or mental issues, such as injuries, infections, psychological traumas, and even death (1). Incidence of dog bites, and their risk factors, have more often been studied in western urban environments. A study conducted in 22 Canadian municipalities between 2003 and 2005 estimated the annual incidence of dog bites to 0–9 per 10,000 inhabitants (2). The incidence is suspected to be underestimated, because they are often underreported by victims who do not always seek medical care (3, 4). The most documented risk factors include age (children) and gender (male) (5–11). Provocative behaviors toward dogs are also often implicated (6–9, 12–15).

In northeastern Canada, more than 75% of the population is composed of Indigenous peoples, including First Nations, Inuit and Métis (16). In the Province of Quebec (Canada), the region located north of the 49th parallel is inhabited by four Indigenous nations: Inuit, Cree, Innu, and Naskapi (17). In these northern Indigenous communities, dogs are known for their historic and important roles, including hunting, transportation and protecting families. However, the presence of free-roaming dogs in small settlements, historical traumas related to the massive culling of northern dogs in the 1950's, the increased use of snowmobiles, and other socio-cultural and environmental changes have complicated the balance between risks and benefits of dogs in those contexts (18, 19).

In northern Indigenous communities, dog bites are frequently reported, but very few studies have investigated their occurrence and risk factors. A recent review showed that available evidence still suggests that Indigenous people living in northern communities are at higher risk of dog bites than the rest of the population (20). However, the occurrence of bites in these communities vary between studies, from 0.61 to 59.6/10,000 annually, with 27 to 62.9% of inhabitants reporting a dog bite during their lifetime (20). In a study conducted in an Inuit community in northern Quebec (Canada), 40.3% of dog owners reported that they, or a family member, had been bitten or scratched by a dog, with a significant higher proportion of dog bites in Inuit people when compared to non-Inuit in the same village (Inuit 62.9% vs. non-Inuit 15.6%) (12). Yet, dog control measures implemented so far lack acceptability. For example, even if dogs are required to be tethered when outside, 78% of the inhabitants reported that they occasionally let their dog roam free (12). Some authors speculated that this lack of acceptability may be due to traditional Inuit practices of letting their dogs loose for socialization and to avoid dog aggression (12, 18).

In northern regions, health risks related to dog bites are exacerbated by the endemic presence of the Arctic Rabies Virus Variant (ARVV) among Arctic fox *Vulpes lagopus* populations in many northern locations, including northern Canada, Alaska, Greenland, Svalbard, and northern Russia (21, 22). In Canada, either in those communities, dog vaccination against rabies is not mandatory. Human death due to rabies is very rare in Canada (25 cases since 1924; the last case being reported in 2012 and due to an exposure in another country) (23). However, the management of dog bites after an incident becomes crucial, as the administration of post-exposure prophylaxis is the only way to prevent the transmission of the rabies virus after a bite. Dog bite management may also be an issue in the northern context for several reasons. Indeed, populations living in northern communities can have different knowledge, attitudes, and practices regarding dog bites and related risks. In a study conducted in an Inuit community in Nunavik (Quebec, Canada), only a minority of people considered themselves at risk of rabies, and 30% reported that they would not consult a health care professional after a dog bite (12). Another study conducted in Nunavik looking in reported dog bites cases from 2008 to 2017 has highlighted that many community members did not complete the rabies post-exposure prophylaxis (PEP) treatment series after their first consultation (8). A study compiling data on the global burden of rabies has also suggested that, in particular contexts, PEP can sometimes be unavailable or not fully completed (24). Moreover, in some communities where by-laws on dogs have been enforced, it has been shown that dog control measures, such as tying up dogs, are inconsistently applied (12) or are sometimes not sustainable over time (6, 25). Finally, health professionals are not always well-prepared for the particular context of these communities and to the different risks to which the populations are exposed. Based on a survey conducted with health professionals working in Nunavik in 2016, 29% did not know any tool (for example public health decision guidelines for PEP administration and bite report form) to use for rabies risk management, and 31% didn't know about zoonotic diseases transmitted by dog bites, except for rabies (26).

To date, dog bites and their management have been studied primarily in the context of Inuit (8, 12, 27), Sahtu (6), Cree and Assiniboine communities in northern Canada (11, 15), and of Alaska Natives from the United States (5, 7). No study has investigated the problem in other northern Indigenous nations, which creates significant challenges in developing locally and culturally appropriate prevention and control programs.

This study aimed to investigate factors that impact the risk of dog bites in the context of two Indigenous nations located in northern Quebec, namely one Naskapi community and one Innu community. A mixed methods study combining quantitative (survey) and qualitative (individual interviews) data was completed to specifically (1) describe the knowledge, attitudes and practices (KAP) regarding dogs and dog bites in these communities, and (2) investigate the experiences of community members and health professionals with regard to dog bites and their management. This study allowed to identify the main barriers to overcome in order to improve current practices, and to implement interventions adapted to the specific context of these communities for the optimal management of dog bites and associated public health risks.

## 2. Methods

### 2.1. Author reflexivity statement

Five authors are non-Indigenous veterinarians living in southern regions of Quebec and one author is an Indigenous Naskapi living in Kawawachikamach. Three of the authors have advanced degrees in epidemiology. The first author (LD) led the data collection, analysis, and manuscript writing. LD is a non-Indigenous woman, a master's degree student in epidemiology and a veterinarian. Five authors were implicated in a systematic scoping review on dog bites in northern Indigenous communities and are familiar with this subject.

### 2.2. Study site

**Figure 1:**
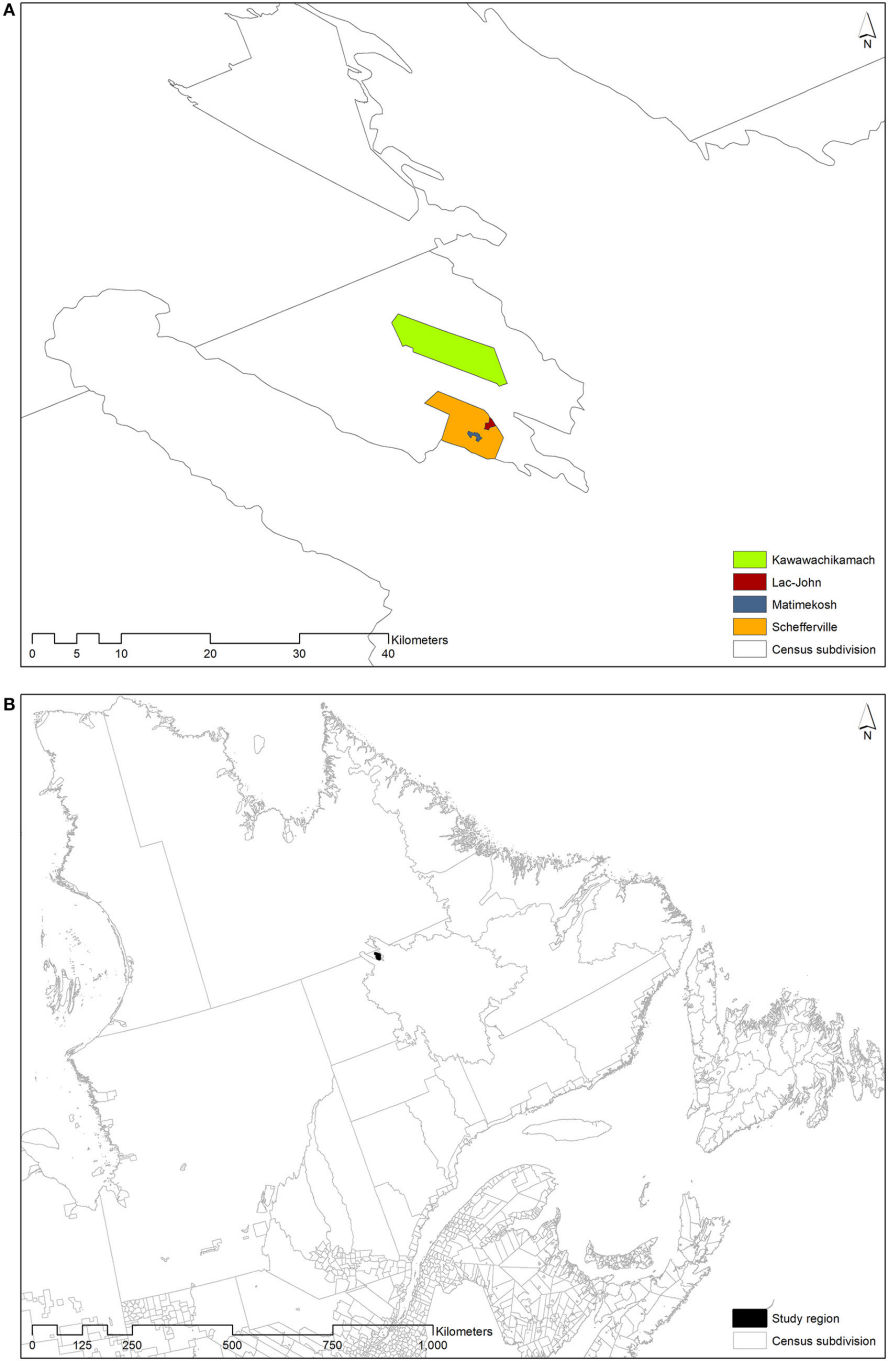
**(A)** Matimekush and Schefferville are adjacent and Lac John is 3.5 km from Matimekush, while Kawawachikamach is located 15 km northeast of Schefferville, near Lake Matemace. **(B)** Those Indigenous communities are located in the province of Quebec (Canada).

This study took place in two Indigenous communities and one administrative municipality located in northern Quebec, Canada: Kawawachikamach (KWW), Matimekush-Lac John (MLJ), and Schefferville (SCH). KWW is the only Naskapi community in Canada and is located 15 km from MLJ and SCH. MLJ is an Innu community surrounded by the territory of SCH (Figure 1A). They are located above the 54th parallel and can only be reached by train or plane (Figure 1B). According to the last census in 2016, the population is 601 inhabitants in KWW (28), 613 in MLJ (29), and 155 in SCH (30). Indigenous people represent 99.2% of inhabitants in KWW (28), 94.3% in MLJ (29), and 48.4% in SCH (30).

Naskapi from KWW are under the James Bay and Northern Québec agreement. This agreement between governments of Quebec and Canada and some Indigenous nations was signed in order to redefine the organization of the territory and its administration (31). Innu from MLJ are under the Indian Act. Only beneficiary Indigenous communities of the James Bay and Northern Québec agreement, either Inuit, Cree and Naskapi, are illegible for the Quebec government vaccination program for northern communities for the protection of dogs against rabies (32).

This study was part of a larger project called *Balancing Illness and Wellness at the Human-Dog Interface in Northern Canada*, which aims to investigate the relationships between dogs, humans and their respective health in Canada, using the “two-eyes” model that combines Indigenous knowledge and Western science. It also aims to propose, implement and evaluate solutions to reduce the risks at the human-dog interface while promoting the positive roles of dogs on human health. The research team received the approval and support of the Naskapi community of KWW, the community of MLJ, and the town of SCH, as part of the global project.

### 2.3. Cross-sectional survey

#### 2.3.1. Sampling and recruitment

The study used a convenience sampling strategy targeting a sample size of 50 adults (over 18 years old) residents in KWW and 50 in MLJ and SCH. Residents were recruited in different places (workplace, grocery store, public places, and some house addresses) until the sample size was reached. The study objectives and the consent form were explained orally and participants were given the choice to complete a questionnaire directly after, on the same day, or later. Naskapi and Innu local coordinators from KWW and MLJ participated in the recruitment of participants and translation of questions when needed.

#### 2.3.2. Data collection

Data collection was mainly conducted in person by the first author (LD) from May 27 to June 12, 2019, in the communities. The questionnaire was based on previous studies and adapted to the current study objectives (see Appendix 1) (12). It included a maximum of 56 questions, with 20 questions restricted to dog owners. The questionnaire was available in French, English, and translated orally in local languages as needed. Questions collected data on: (1) dog demography (male or female, breed, reproductive status, age, roles, vaccination status, time spent free-roaming), (2) veterinary services available in the community and those that would be desired (results for this part are not presented and are available in Appendix 2), (3) experiences with dog bites (themselves or in their surroundings, context, and actions taken after), (4) perceptions of dogs and situations related to dogs (knowledge on rabies, perceived susceptibility of being bitten or contracting rabies in the community, perceived severity and level of concern related to dogs, rabies, and dog bites), and (5) demographic data on the participants (age, gender, occupation, beneficiary of the James Bay and Northern Quebec Agreement). Questions pertaining to perceptions were evaluated using a five-point Likert scale. For the questions specific to dogs, the respondents could give information for a maximum of four dogs (four older dogs owned). At the end of the questionnaire, people were invited to participate in an individual interview.

#### 2.3.3. Data analysis

Data were compiled and analyzed using IBM SPSS Statistics (RRID:SCR_016479) version 25 software. Because of the proximity between MLJ and SCH, the low number of respondents, the culture difference and the different access to government dog rabies vaccination program, data from both MLJ and SCH communities were compiled (MLJ-SCH) and compared to data from KWW, meaning that two localities were compared. Descriptive analyses were conducted globally and by the community. Statistical tests were performed to assess significant differences in proportions between communities with *p* < 0.05, with either Pearson's Chi squared test or Fisher exact test when the theoretical size of any cell was lower than 5. All significant results were reported in the result section. Missing data were excluded to calculate proportions for individual variables.

An exploratory factor analysis (EFA) was used to explore the underlying structure of perception variables and to identify variables to regroup for further analysis (33). It was initially performed on the fifteen risk perception variables. As it is generally recommended for ordinal psychosocial data, we used the unweighted least square extraction method and an oblimin rotation (34, 35). The correlation between variables was assessed by Pearson correlation. The quality of representation was assessed by initial communalities and results inferior to 0.2 were excluded from the analysis. We excluded factors with eigenvalues below 1 and we included variables with factor loadings superior to 0.5. Sampling adequacy was verified by calculating the Kaiser-Meyer-Olkin (KMO) value and Bartlett's Test of Sphericity, for each latent factor. *P*-values < 0.05 were considered statistically significant. For each participant, the factor scores of latent variables were estimated by summing the initial scores corresponding to all items loading regrouping on a factor, as it is generally accepted for exploratory analysis (36, 37). New variables were created with these sum scores: Dog risk perception, Rabies risk perception and Perceived ability to protect oneself against rabies. Cronbach's alpha was calculated, as evidence for the reliability of the measurement and a value of 0.65 and above was deemed acceptable (38).

Multivariable regressions were used to explore the association between selected factors and three outcome variables: (A) knowledge on rabies (high vs. low reported knowledge, binary logistic multivariate regression); (B) dog risk perception (linear multivariable regression of the discrete perception score); and (C) exposure to dog bites during the lifetime (yes vs. no, binary logistic multivariable regression). The dichotomized variable “Knowledge on rabies” was created by combining, “Never heard of rabies” and “Little knowledge” (low), as well as combining “Basic knowledge” and “Extensive knowledge” (high). To build the models, we used at first univariable regressions for each independent variable to assess their association with the dependent variable: age, gender, knowledge on rabies (except for model A), communities, owning dogs, been bitten (except for model C), Dog risk perception (except for model B), perceived ability to protect oneself against rabies, and Rabies risk perception. Variables associated to the outcomes with *p* < 0.2 were kept for inclusion in the multivariable models. Then, multivariable models were built using a backward elimination process with *p* < 0.05. Gender, age, and communities were forced into all models, significant or not, in order to include the factors considered potentially confounding. Interactions were also assessed between some variables (gender, age, and community). For model C using exposure to dog bites as the dependent variable, there was no significant association with all tested variables, and so results for this model are not presented (available in Appendix 3).

### 2.4. Individual interviews

#### 2.4.1. Sampling and recruitment

We aimed to recruit around 30 participants (15 in KWW and 15 in MLJ-SCH) in order to achieve data saturation and capture all perspectives on different questions and to identify common themes (39). Participants represented three categories of community members (Indigenous or not; over 18 years old): (1) residents who have had a dog bite before (or parents of children who have had a dog bite), (2) residents who owned a dog that has bitten a person before and (3) health care professionals involved in dog bite management. Residents were invited to participate when they completed the questionnaire. The study objectives and a new consent form specific for interviews were explained orally, and oral or written consent was obtained for all participants before interviews. Qualitative data was collected after quantitative data for all participants, and all interviews were conducted between May 27th and June 12th, 2019. Naskapi and Innu local coordinators from KWW and MLJ also helped with the recruitment of interviewees and translations when needed.

#### 2.4.2. Data collection

Semi-structured interviews were conducted by a team member (LD) in French or in English and lasted between 8 and 43 min (mean: 20 min). The interview guide was developed by the research team in collaboration with the local community coordinators (see Appendix 1). Subjects addressed their experiences with dog bites, their perception of rabies risks and their perceptions of how to improve services regarding dogs and dog bites. Subjects discussed with health care professionals also included their roles in dog bites and dog transmitted zoonosis management. All interviews were audio recorded. In order to maintain participants' confidentiality, localities of the nurses interviewed will not be mentioned in the results section.

#### 2.4.3. Data analysis

Recordings were transcribed verbatim with the help of a professional transcriber and imported into the software NVivo (RRID:SCR_014802) version 12.6 for analysis. Thematic analysis was used to analyze the transcripts. One member of the study team (LD) read through all interview records repeatedly to become familiar with the transcripts, and CA read a sample of them. Themes and codes were generated using an inductive approach to capture the salient features of the data and to represent the main topics raised. Code development was made according to the conceptual knowledge of the research team based on a review of the literature (20) and the interview material. Codes were initially developed independently by LD and CA and an interactive discussion followed to get a consensus on the code set. Codes were applied on the different transcripts, and a rearrangement of the data was made to analyze the content according to codes.

### 2.5. Integration of quantitative and qualitative data

Quantitative and qualitative data were mixed following a triangulation design. Qualitative data were used to integrate Indigenous knowledge and perspectives. The integration of the results was done according to a multilevel model, e.g., qualitative data were collected only from a subgroup of respondents who completed the quantitative questionnaires. Both types of data had equal weight at the level of analysis and interpretation. They were first analyzed separately and then contrasted, in order to have a complete conclusion on the study objectives, either the KAP regarding dogs and dog bites in these communities and the investigation of experiences of community members and health professionals with regard to dog bites and their management (40).

### 2.6. Ethical committee approval and consent

A member of one of the targeted communities participated in verifying the questions asked in the survey. Band councils were also notified of the data collection and were consulted on recruitment methods. Both consent forms for interviews and survey, available in French and English, were filled beforehand or given orally, which is a procedure approved for an Indigenous context by the ethical committee. Indeed, oral consent is an appropriate alternative to obtaining written consent in a way to respect the aspect of traditional oral information transmission (41). The participants of the questionnaire were invited to participate in a draw that gave them the chance to win a prize worth CAD 50$. The participants who also completed an interview all received a financial compensation of CAD 25$. All participants (from surveys and interviews) were invited to participate in a draw for a chance to win one of three material prizes (dog food and dog accessories). Each host community received a dog cage (value of ~CAD 300$). The project protocol was reviewed and approved by the ethical committee at the Université de Montréal (Comité d'éthique de la recherche en sciences et en santé; certificate number #CERSES-19-048-P). A feedback on the results will be available to the communities.

## 3. Results

One hundred and twenty-two people completed the survey (KWW: 56; MLJ-SCH: 66). Two thirds were women, approximately one third were between 18 and 29 years old, another third were over 49, and most identified themselves as Indigenous (Innu: 41%; Naskapi: 34%; Non-Indigenous: 7%; Table 1). The respondent age distribution matched the age distribution based on the last census in 2016 (28–30) (see Appendix 4).

**Table 1:** Sociodemographic description of the survey participants compared between study regions (*n* = 122).

	**KWW**	**MLJ-SCH**	**Total**
	***n* (%)**	***n* (%)**	***n* (%)**
**Total**	**56**	**66**	**122**
**Gender**			
Women	30/51 (59%)	46/63 (73%)	76/114 (67%)
Men	21/51 (41%)	17/63 (27%)	38/114 (33%)
**Age (years old)**			
18–30	17/49 (35%)	21/61 (34%)	38/110 (35%)
30–39	9/49 (18%)	12/61 (20%)	21/110 (19%)
40–49	8/49 (16%)	8/61 (13%)	16/110 (15%)
More than 49	15/49 (31%)	20/61 (33%)	35/110 (32%)
**Time spent in the community (years)**			
<11	13/46 (28%)	6/60 (10%)^*^	19/106 (18%)
11–30	17/46 (37%)	22/60 (37%)	39/106 (37%)
31–50	13/46 (28%)	17/60 (28%)	30/106 (28%)
>50	3/46 (7%)	15/60 (25%)^*^	18/106 (17%)
**Indigenous nation^α^**			
Naskapi	37/56 (66%)	4/66 (6%)^***^	41/122 (34%)
Innu	0/56	50/66 (76%)^***^	50/122 (41%)
Naskapi and Innu	1/56 (2%)	4/66 (6%)	5/122 (4%)
Non-indigenous	4/56 (7%)	4/66 (6%)	8/122 (7%)
Other or unknown^β^	14/56 (25%)	4/66 (6%)^**^	18/122 (15%)

α The effective of the cells is lower than 5. A Fisher test has been executed between each category and the rest.

β Other includes people that are both from the Naskapi and Innu nations or people from other Indigenous communities.

* *p* < 0.05,

** *p* < 0.01,

*** *p* < 0.001.

Thirty-seven people completed the interview (KWW: 18; MLJ-SCH: 19), including 23 people reporting a dog bite (KWW: 10; MLJ-SCH: 13), two who owned a dog that had bitten a person (KWW: 1; MLJ-SCH: 1), and 12 health care professionals (KWW: 7; MLJ-SCH: 5). Most identified themselves as Indigenous (Innu: 24%; Naskapi: 41%; other Indigenous nations: 10%).

### 3.1. Knowledge on dog bites and rabies

Seventy-three percent (85/117) of survey respondents did not know that they were at risk of contracting rabies after a dog bite. Also, 50/116 (43%) of respondents described themselves as having at least basic or extensive knowledge about rabies. In the multivariate model, age was the only variable associated with Knowledge on rabies. The odds of a higher knowledge on rabies were 2.924 higher (*p* < 0.05; CI 1.071–7.979) in the 18–29 age group than the 50 and more group (Table 2, A).

**Table 2:** Factors associated with (A) knowledge on rabies and (B) dog risk perception (*n* = 106) from multivariable regressions.

**(A) Knowledge on rabies [logistic regression of the dichotomized scores (high vs. low)]**		
	**OR**	**95% CI**
Community (KWW: ref)	0.635	(0.277–1.454)
Gender (Woman: ref)	1.504	(0.621–3.638)
Age (50+ yr: ref)		
18–29 yr	2.924^*^	(1.071–7.979)
30–39 yr	0.364	(0.098–1.349)
40–49 yr	1.532	(0.451–5.197)
**(B) Dog risk perception (linear regression of the discrete perception score)**		
	**Coefficient**	**95% CI**
Community (KWW: ref)	−0.432	(−1.731–0.867)
Gender (Woman: ref)	−0.474	(−1.850–0.901)
Age (50+ yr: ref)		
18–29 yr	−0.057	(−1.664–1.550)
30–39 yr	−0.102	(−1.948–1.744)
40–49 yr	−0.580	(−2.598–1.438)
Rabies risk perception	0.691^***^	(0.363–1.018)

* *p* < 0.05,

*** *p* < 0.001.

Similar lack of awareness was also reported by most interviewees when asked if community members were well-informed about dog bite risks: “*I don't think so. Nobody really cares*” (ID025—KWW). There was also a global lack of knowledge on rabies, as one interviewee mentioned: “*According to* [people in the community]*, It* [rabies] *must be just a disease of dogs, but they must not even know that it is transmitted to humans. Is it transmitted to humans?*” (ID018—MLJ-SCH, translation).

### 3.2. Attitudes and risk perception

Most participants [76/115 (66%)] agreed that dogs can transmit disease and most participants [74/113 (65%)] perceived that dog bites are a serious health problem. The risk of being bitten by a dog in general was also recognized as high by participants: 66/116 (57%) agreed, whereas 30/116 (26%) were unsure. Descriptive analysis of perceptions is available in Appendix 5.

**Figure 2:**
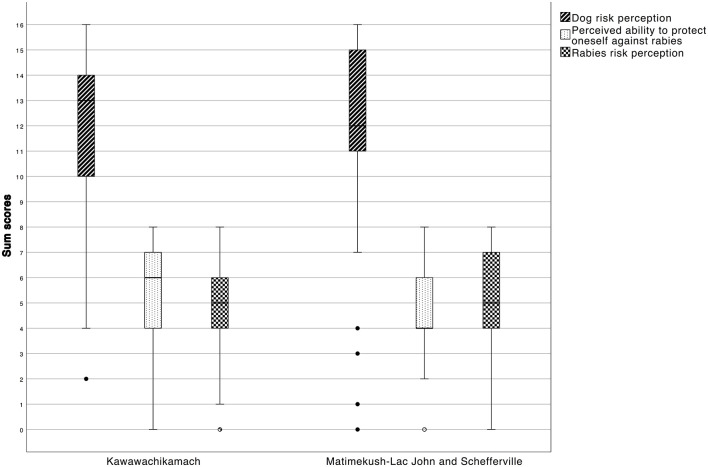
Box plot distribution of summation score for the three latent factors of the EFA, for each locality.

The final exploratory factor analysis (EFA) with eight variables suggested three latent factors that were named: Dog risk perception, Perceived ability to protect oneself against rabies, and Rabies risk perception. Initial and final EFA with factor loadings are presented in Appendix 6. The final model explained 57.8% of the variance. Cronbach alpha was 0.758 for Dog risk perception (four variables: There are too many dogs in your community, Dogs can transmit diseases, Dog bites are a serious health problem and The risk of being bitten by a dog in or around my community is high), 0.836 for Perceived ability to protect oneself against rabies (two variables: It is easy for me to protect myself against contracting rabies and It is easy for me to protect members of my family against contracting rabies), and 0.668 for Rabies risk perception (two variables: The risk of contracting rabies in or around my community is high and I am worried that you or one member of your family are at risk of contracting rabies). The distribution of sum scores of these three factors are presented in Figure 2.

In the final multivariable models, Rabies risk perception was positively associated with Dog risk perception (Table 2, B). Communities and gender were not significantly associated with Dog risk perception.

In the survey, a majority of respondents of both localities [100/116 (86%)] perceived that there were too many dogs in their community. This was also reflected in the interviews: “*Yeah, there are too many dogs and there is no service here about controlling the dogs or the dog's population or whatever*” (ID021—KWW). Worries about packs of dogs were reported, especially for children playing outside: “*Because you have dogs running around and dogs forming packs, so I feel like that's more dangerous, especially for kids because we have a lot of kids that are outside even small kids and I feel like they're not being watched closely*” (ID025—KWW).

The level of fear of dogs differed among survey respondents in both localities, with 65/116 (56%) of the survey respondents who disagreed with the statement that they feared dogs in their community (no significant difference between communities).

Interviews revealed a diversity of feelings regarding dogs in the community. Some people reported that they had developed a fear of dogs, while others mentioned being comfortable with free-roaming dogs, even if, in some cases, they have been bitten by a dog before. One interviewee has been afraid of dogs since her dog bite: “*All my life, I just hear a yelp... then all my nerves are awoken* […] *At one point, I walked around with a Sling shot, a little sling shot*. […] *I never used it, it was just a security you know*” (ID001—KWW, translation). Some of the interviewees reported that they were particularly uncomfortable with free-roaming dogs. One nurse reported that “*there are many patients who are afraid of dogs and they often come to the dispensary with a stick*” (ID013—Nurse, translation).

When present, the fear of dogs had important consequences on quality of life of certain participants: “*I had a dog phobia at one point*. […] *I was not able to go see my grandparents, me who was so close to my grandmother and my grandfather. I didn't see my grandfather for 2 or 3 weeks after* [the dog bite event]” (ID006—MLJ-SCH, translation). It was also reported as a barrier to physical activity for one of the interviewees: “*They are free roaming here. I can't take a walk*. […] *I take my pick-up to go somewhere. I would like it to walk, but I'm too afraid of dogs*” (ID024—MLJ-SCH, translation). One of the parent interviewees was afraid of letting her children play outside alone.

On the other hand, it was mentioned that free-roaming dogs were still perceived as protectors against wildlife: “*I think it is very good to have dogs around here. They give you alert, if something happens, like outsiders, animals*. […] *If you hear dogs barking outside the community, you know there is an animal*. […] *Because kids are wandering everywhere*” (ID035—KWW). A few interviewees mentioned that dogs can protect them when they are walking in the community or go out in the land at their camp outside the community: “*Every time I go home at night, I have one with me and I feel safer because of it, if there is an animal around, I might use some help*” (ID028—KWW).

### 3.3. Practices regarding dogs

Among the survey respondents, 47/122 (39%) were owners of one or more dogs. The majority [38/47 (81%)] of those owners had only one dog, for a total of 28 dogs owned in KWW and 42 dogs in MLJ-SCH (Appendix 7). The principal role of the dogs was as companions [8/13 (62%) in KWW and 31/35 (89%) in MLJ-SCH; *p* < 0.05)]. There were 6/9 (67%) of dogs from KWW and 29/30 (97%) of dogs from MLJ-SCH whose owners reported that they had been vaccinated in the last 12 months (*p* < 0.05).

Interviewees also described dogs as members of the family and sometimes as members of the community: “*People like dogs a lot even if it's not your dog. You know from which family he comes from, I mean… Because we all know each other here*” (ID002—MLJ-SCH, translation). One interviewee perceived changes in the status and role of dogs over time: “*if I go back 20 maybe 30 years ago, this is a discussion from the elders, dogs were a priority in the community. They were for hunting, fishing, you name it. But things have changed when they had dogs in every household*. […] *if you go back 50 years ago, when they lived up in George River, they knew what the role was. You take care of your dog because it's your life support. Today, the new generation, it's not your life support, it's just—it's a pet*” (ID016—KWW).

In the survey, 14/15 (93%) of owners in KWW and 13/29 (45%) in MLJ-SCH let their dogs live outdoors (*p* < 0.01). 30/44 (68%) of total owners reported letting their dogs free roam occasionally (Table 3). Interviewees' opinions on whether they would like dogs to be tied up or not were heterogeneous. Some interviewees perceived this practice as necessary: “[a suggestion/idea] *maybe more for the owners of the dogs, to tie up their dogs and take care of their dogs if they're going to have dogs and take care of them properly, not leave them running around like that*” (ID015—KWW). However, many interviewees reported that tying dogs also had negative consequences for their health and wellbeing: “*I think you should be walking your dog like habit exercise and then, maybe, if you're comfortable, bring it inside, like how you would treat a family, but at night, let tied it up so it's just safe in its dog house. I don't think they should be tied up all the time. I think it's another form of abuse*” (ID031—KWW). Some people have also reported that this practice may increase dog aggression in some context: “[…] *I know some people that just tie up their dogs indefinitely and they get dangerous over time because they're so secluded to their environment, they need to roam around, but maybe they should—they should put a leash on them, walk with them, play with them, love them*” (ID027—KWW). It was also normal for some interviewees to see dogs roaming free: “*Well, I'm actually comfortable. It has been like that for a long time*. […] *Like in the cities there are stray cats. Here, there are dogs*” (ID028—KWW).

**Table 3:** Practices of owners regarding the management of their dogs.

	**KWW**	**MLJ-SCH**	**Total**
	***n* (%)**	***n* (%)**	***n* (%)**
**Dog is kept:**			
Mostly indoors	1/15 (7%)	10/29 (34%)	11/44 (25%)
Indoors and outdoors	0/15	6/29 (21%)	6/44 (14%)
Mostly outdoors	14/15 (93%)	13/29 (45%)^**^	27/44 (61%)
**Is mostly outdoors, it is**			
Free roaming	3/15 (20%)	11/29 (38%)	14/44 (32%)
In a pen	2/15 (13%)	1/29 (3%)	3/44 (7%)
Tied up	7/15 (47%)	15/29 (52%)	22/44 (50%)
Both tied up and free	2/15 (13%)	2/29 (7%)	4/44 (9%)
Other	1/15 (7%)	0/29	1/44 (2%)
**I let my dog roam free occasionally**	9/15 (60%)	21/29 (72%)	30/44 (68%)

** *p* < 0.01.

Practices toward dogs were criticized by many interviewees, Indigenous and non-Indigenous. One Innu interviewee reported that practices regarding dogs were different for Indigenous people: “*And people, they let their dogs roam and they don't keep them like the non-Indigenous*. [The non-Indigenous], *they keep their dogs at home, they have chains and* […] *leashes. But, here, it seems as if there is a letting go*” (ID012—MLJ-SCH, translation). A few interviewees reported specifically that it is important for themselves to take good care of their dogs: “*Yeah, because I think dogs are good loyal friends if you take care of them*” (ID030—KWW).

### 3.4. Experiences with dog bites and bite management

Among respondents, 23/112 (21%) reported a dog bite in their lifetime (Table 4). Twelve (12/22; 55%) reported bite events occurred when the dog was roaming free. Otherwise the dog was tied up, on a leash or other (Table 5). Twelve (12/22; 55%) also consulted a health care professional after their dog bite.

**Table 4:** Proportion of respondents reporting at least one dog bite in their lifetime, whether the victim is themselves, other adults or children of their surroundings, in KWW and MLJ-SCH (*n* = 112).

	**KWW**	**MLJ-SCH**	**Total**
	***n* (%)**	***n* (%)**	***n* (%)**
Respondent	13/50^α^ (26%)	10/62 (16%)	23/112 (21%)
Adult in the surroundings	11/50 (22%)	14/62 (23%)	25/112 (22%)
Child in the surroundings	19/50 (38%)	10/62 (16%)^**^	29/112 (26%)
In the last 12 months^α^	9/32 (28%)	5/25 (20%)	14/57 (25%)

** *p* < 0.01.

α Participants could answer either the bite was on themselves and either if they knew someone (adult or child) from their surroundings that had a dog bite. This is why the total of each category does not correspond to the denominator.

β All bites were considered for this question. If more than one option was selected for the previous question, the most recent bite was considered. Only two people who answered that the bite was on them mentioned that it was in the last year (000019 and ID_P018). However, they also ticked that they knew someone bitten.

**Table 5:** Contextual factors and behaviors related to dog bites (based on the 22 survey respondents who reported a dog bite).

	**KWW**	**MLJ-SCH**	**Total**
	***n* (%)**	***n* (%)**	***n* (%)**
**Know the dog that bit you**			
No	5/12 (42%)	5/10 (50%)	10/22 (45%)
Dog of someone from my surroundings	5/12 (42%)	5/10 (50%)	10/22 (45%)
My dog	1/12 (8%)	0	1/22 (5%)
Other	1/12 (8%)	0	1/22 (5%)
**Was the dog who bit you:**			
On leash	1/12 (8%)	1/10 (10%)	2/22 (9%)
Tied-up	4/12 (33%)	2/10 (20%)	6/22 (27%)
Free roaming	5/12 (42%)	7/10 (70%)	12/22 (55%)
Other	2/12 (17%)	0	2/22 (9%)
**After bite, done:^†^**			
Nothing	3/12 (25%)	3/10 (30%)	6/22 (27%)
Rinsed/cleaned wound	2/12 (17%)	2/10 (20%)	4/22 (18%)
Disinfected	1/12 (8%)	0	1/22 (5%)
Put a bandage	1/12 (8%)	2/10 (20%)	3/22 (14%)
Consulted a nurse/doctor	5/12 (42%)	7/10 (70%)	12/22 (55%)
Consulted another member	1/12 (8%)	0	1/22 (5%)
Killed the dog	0	1/10 (10%)	1/22 (5%)
Contacted a member to kill dog	1/12 (8%)	2/10 (20%)	3/22 (14%)
Other	4/12 (33%)	1/10 (10%)	5/22 (23%)
**Consult a health professional after bite**	5/11 (45%)	7/10 (70%)	12/21 (57%)
Get a rabies shot	5/5 (100%)	5/7 (71%)	10/12 (83%)

† One respondent could answer more than one question.

Interviewees reported different levels of awareness regarding what they needed to do in case of a dog bite. The majority of interviewees mentioned that they seek health care after their bite: “*Yes, they know* [generally what to do after being bitten], *they go to the dispensary*. […] *they say, “OK, go to the dispensary, it's dangerous, you're going to get an injection there. A vaccine to not get sick.” Often people will go there*” (ID002—MLJ-SCH, translation). However, six interviewees reported that they would not know what to do: “*They tell us what to do when there's a fire, but they could also tell us what to do when there's a bite*” (ID001—KWW, translation). Finally, some interviewees mentioned that they did not consult any health professionals after their bites: “[After my dog bite I did] *nothing*. […] *I didn't report it. I never report it*” (ID021—KWW). Few interviewees mentioned that, being bitten as a child, they were afraid to tell their parents about their dog bites: “*I didn't tell my parents, I just went home. No blood*” (ID019—MLJ-SCH). Nurses from both communities also reported difficulties in follow-ups after the first visit: “*But sometimes they will come because there is a wound, to see if it is infected or not. But after that, it's more at the follow-up level that we have problems*” (ID005—Nurse, translation). Another nurse also reported: “*So often they don't show up or they don't feel the importance of even though they're told about rabies and that it's deadly and everything*” (ID011—Nurse, translation).

In the survey, 19/50 (38%) from KWW and 10/62 (16%) from MLJ-SCH knew a child from their surroundings that has been bitten in their lifetime (*p* < 0.01). Several interviewees also mentioned children being at higher risk of bites than the rest of the population: “*Most of them are children who get bitten*” (ID004—Nurse, translation). Provocative behaviors were sometimes mentioned explaining this risk: “*They* [children] *are throwing out rocks. And my dogs can be pissed and be mean back to you. They throw rocks at him*” (ID035—KWW). However, other cases reported by some interviewees seem unprovoked by the child involved: “*She said that the dogs were biting at her while she was trying to walk toward the community center and then she would try to push them away or try to scare them away*” (ID027—KWW).

All nurses mentioned having a clear and specific protocol to follow in a case of dog bites: “*I learned it by doing it and the protocol is really clear, basically*” (ID011—Nurse, translation). Most reported having learned specifically about zoonosis through their work experience, rather than during their formal training: “*I don't really have* [any training in rabies and zoonoses]. *We have procedures. I can show you the procedure to follow when there are dog bites. Then everything is detailed*. […] *It's done on the job. We are always 4* [nurses]*, then there is always an old one like me there. Then who says…/ But all the procedure manuals are there, clearly identified*” (ID004—Nurse, translation).

Several challenges were reported with regard to the management of biting dogs. First, interviewees mentioned that it was sometimes difficult to identify and find the dog following an event: “*We try to find the owner of the dog, first. And it's not an easy thing so we have cooperation from the police because there are a lot of stray dogs*. […] *Since we don't have an owner's name, we can't find the owner*. […] *So, I gave* [the public health] *the contact for the Naskapi police*” (ID004—Nurse, translation). Second, there was confusion about roles and responsibilities with regard to biting dogs: “*I know the* [Local health care center] *want us to pick up the dog and bring the dog to the* [Local health care center], *we can't do that*. […] *Yeah, and the thing is we didn't even have a policy on that. I could show you, we have two big binders on policies, on crime you name it, but nothing on dogs. Cause usually it is not a policing problem. Most communities I work in, we have a dog control officer that deals with it*” (ID016—KWW). Finally, interviewees mentioned that there are no assigned areas to quarantine dogs after a dog bite, which creates confusion as no one feels responsible for managing the period of observation of the biting dog.

Most interviewees mentioned that biting dogs are generally killed after an incident: “[…] *when there is a dog bite, they have to kill the dog because he already has a taste for it, you know, the blood. Yeah, it was sad*” (ID030—KWW). Some people also mentioned that biting incidents sometimes led to dog culling in the community: “*And after this incident, all the dogs had to be put down except the small ones, I think. And people were really mad in the community that they went that far*” (ID030—KWW).

## 4. Discussion

This mixed methods study allowed to describe and compare the knowledge, attitudes and practices (KAP) regarding dogs and dog bites in two Indigenous communities; Naskapi from KWW and Innu from MLJ.

**Figure 3:**
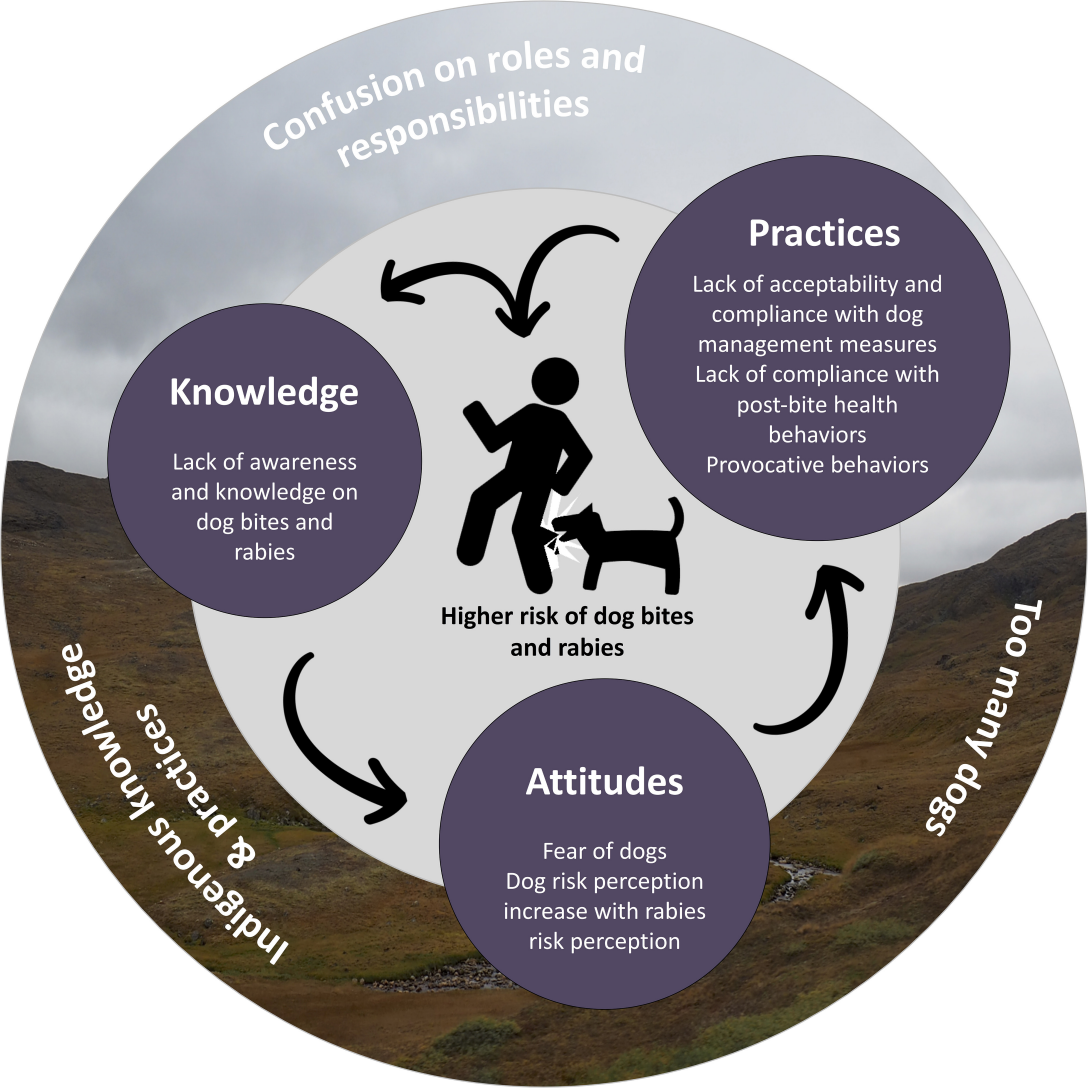
Main factors influencing the risk of dog bites and rabies in the context of KWW and MLJ-SCH communities [adapted from Daigle et al. (20)]. Furthest factors are contextual and environmental factors and circles in the middle are illustrating the main individual factors.

Main factors increasing the risk of dog bites and rabies that emerged from this study are synthesized in Figure 3. Most of these factors have also been reported in previous studies conducted in other Indigenous northern communities (20) and they are known to interact with the adoption of practices. For example, knowledge and risk perception are known to be determinants of health behaviors in several theoretical models such as the Health Belief Model and the Theory of Planned Behavior, and are well-documented factors that affect compliance with preventive practices (42, 43). All these factors need to be considered in the development of effective and acceptable preventive programs for dog bites and rabies in Indigenous northern communities.

Specifically, this study revealed a lack of awareness and knowledge about dog bites and rabies risks in both communities. This is not the first time that a lack of knowledge on those risks is reported in studies addressing dog bites risks. Indeed, a survey among 5–15 year olds consulting for dog bites in United States pediatric emergency departments reported that 42% of children did not pass a test on dog bite prevention knowledge (44), demonstrating that in young people from the general population, knowledge of dog bites is considered low. Another study on the Navajo Reservation, in 1986, also noted the importance of public education, especially for children and parents (45). Education measures such as learning about basic dog behaviors and ways to avoid interactions leading to dog bites have been discussed (45).

Results showed that the role of dogs is changing in the studied communities, which is coherent with observations from studies conducted in other northern Indigenous communities (15). In previous research on Inuit dogs and their relationships with people in the Canadian Eastern Arctic, dogs were used mainly for hunting and transportation as an integral part of the economy of Inuit, until the middle of the twentieth century (18). This particular role of transportation has been replaced by other transportation modes, even if the symbolism of dogs is described as still existing (18). This shift of needs from dogs is suspected to change the importance of how dogs are perceived; it is not a “life support” anymore, as an interviewee mentioned in this study.

If free-roaming dogs are still perceived as protectors by community members, our study also suggests that too many free-roaming dogs can create fear for some people, which in turn can impact behaviors and quality of life, for example by preventing physical activity in communities. This observation has been reported in a study conducted in 2011 among teenagers in American Samoa, an Indigenous community located in a United States territory in the South Pacific, where 14% of them mentioned that fear of dogs prevented them from getting more exercises (46). Psychological consequences of dog bites have been reported in other contexts. In a survey conducted in New Zealand, 72% of respondents reported psychological consequences after a dog bite, going from short-term and minor effects, to long-term and severe consequences requiring, for example, counseling (47).

A topic that was frequently discussed by participants and on which there was no consensus was whether a dog should be tied up or free-roaming, demonstrating the complexity of this issue. Free-roaming dogs can be owned or ownerless. Another study conducted in an Inuit community in 2018 underlined the importance for some inhabitants of letting dogs roam free (12). In a study conducted in an Indigenous community in the United States, it was even expected by some inhabitants that dogs follow and protect children (48). This study highlights that the “prototypical” dog on the reservation lives differently from the “contemporary American prototypical” dog; the concept of living freely and having autonomy within the community is noted by authors (48). This complexity should be considered in the development and implementation of measures for reducing dog bites risks. Strategies to control dog populations need to consider the duality between liberty concepts comparatively to restrictions notions, in order to be acceptable and durable. Also, there was a significant difference between community owners for letting their dogs live outdoors (higher proportion in KWW), emphasizing that local context is important. It is not possible to explain this difference according to the results.

In this study, Rabies risk perception was a factor influencing the level of risk perception toward dogs (Dog risk perception) in the communities, even though there was a reported lack of knowledge on rabies. This study also showed that age was associated with knowledge on rabies, with young adults being the group with the highest knowledge on rabies when compared to those aged 50 and over. This observation suggests that older adults should also be considered when designing education programs on dogs and rabies. In a 2021 study on interactions between children and dogs in an Inuit village (Canada), it was suggested that education programs on dog bites and care should target adults to raise their awareness and consequently, increasing child supervision in regard to dogs (49).

The proportion of dog bites over the lifetime estimated in this study was slightly lower than what was found in previous research conducted in other Northern Indigenous communities (21% of study participants vs. 27 to 62.9% as reported in a recent systematic scoping review) (20). Risk factors for dog bites that are well-documented in literature, including gender and age, were not significantly associated with dog bites in this study, although the higher risk of children was reported regularly during the interviews.

Nurses from both localities reported that they were adequately prepared to manage dog bites and risks related to rabies, which is good news from a public health perspective. A major issue that came out from interviews was that a significant proportion of people do not seek medical care after a bite, which is of concern for public health. Awareness should be raised among the general population in order to emphasize the risks of not consulting, and the best practices to adopt after a dog bite.

In addition, confusion about roles and responsibilities regarding the management of the biting dog may impair the capacity to prevent consequences following a bite. Killing the dog was sometimes implemented after a bite. This practice has been reported in similar contexts (12, 50). However, killing the biting dog can complicate the post-bite observation of dogs, which is an important measure for rabies prevention. Inconsistency or limited application of animal control services was also noted in previous studies (6, 8, 25). In a survey investigating perspectives from users of veterinary and dog services in remote, rural, and Indigenous communities of northern Manitoba, all participants mentioned a need for a dog-related by-law officer for addressing issues with dogs and to enforce dog legislation (51). This underlines the urgent need to clarify these responsibilities among local stakeholders at the community level.

This study was exploratory and had limitations. This study used a cross-sectional design which limits the evaluation of the temporality between risk factors and bites. Future studies should consider cohort design in order to obtain incidence measures and to estimate other associations with risk factors. Then, a convenience sampling strategy was chosen to reach a sufficient number of participants in each community. This sampling strategy could lead to a lack of representativeness of the sample. However, we did compare the distribution of age and gender between our sample and the census data and concluded that the representativeness was good (Appendix 4). Also, it was discussed with community members as the most acceptable way of administrating questionnaires. The sample size was estimated according to calculation, based on the population size. For the qualitative portion of the study, we only interviewed adults, which restricts our understanding of factors of importance in children. Still, parents of children who have had a dog bite were interviewed. Given the over-representation of children in dog bite cases, this could be better investigated in future studies.

The small sample size restrained our capacity to detect significant association between factors and outcomes investigated in this study. We chose this sample size because this study was exploratory and we wanted to avoid over-solicitation from researchers, a common problem in Indigenous communities (52). However, this research protocol was discussed and approved by community band councils. The fact that these are small communities gives an advantage in terms of representativeness and internal validity, because a small sample could represent an adequate proportion of the source population. Lastly, dog bites in this study were self-reported by participants based on their lifetime, which can limit the interpretation and the comparison with annual incidence data in other studies, and can lead to recall bias in minor dog bite cases.

This study was the first to investigate dog bites in a Naskapi community and an Innu community. It provides important knowledge on risk factors related to dog bites in northern Indigenous communities, and it delivers a first report on how dog bite management is experienced by inhabitants and health care professionals. The mixed methods design made it possible to deepen knowledge on dog bites and rabies KAP and to integrate Indigenous knowledge and perspectives. This study revealed a lack of awareness and knowledge about dog bites and rabies risks in both studied Indigenous communities. Results provide important knowledge for the development of interventions adapted to the particularities of northern Indigenous communities.

## Data availability statement

The raw data supporting the conclusions of this article will be made available by the authors, without undue reservation.

## Ethics statement

The studies involving human participants were reviewed and approved by Comité d'éthique de la recherche en sciences et en santé at the Université de Montréal; certificate number #CERSES-19-048-P. The patients/participants provided their oral or written informed consent to participate in this study. The CERSES approved the use of oral consent for participants whenever it was not possible to obtain written consent. First Nations and Inuit communities primarily populate the northern regions in which this project is taking place. As described in the First Nations of Quebec and Labrador Health and Social Services Commission's Principles of Research Toolkit, members of First Nations' communities may express fears about signing forms, and it is essential to establish a relationship of openness and trust in research. In order to accommodate these cultural differences, our committee accepts the recording of oral consent from participants. This practice is consistent with the requirements of the Tri-Council Policy Statement on Ethical Conduct for Research in Canada.

## Author contributions

LD: study design, data collection, analysis, and interpretation of data and redaction. AR and CA: study design, contribution to the data analysis and interpretation, and revision of the manuscript. YR, AS, and KM: contribution to result interpretation and final revision of the manuscript. All authors contributed to the article and approved the submitted version.
