# Supplementary material for: Knowledge, attitudes, and practices regarding dogs and dog bites in Indigenous northern communities: A mixed methods study
Source: Front Vet Sci. 2023 Feb 20;10:1080152. doi: 10.3389/fvets.2023.1080152 (PMC9986472; doi:10.3389/fvets.2023.1080152)
Supplement: Supplementary file 1 [file Data_Sheet_1.PDF]

Dogs survey

The purpose of this survey is to collect information about how dogs are perceived in your communities and the health risks that may be related. The goal is to better understand and respect your perspective and needs in order to implement interventions and services for you and your dogs.

Your participation is very important to us. Completing this survey will take between 5 to 10 minutes of your time. Questions are mainly multiple choice.

Your participation is voluntary. Therefore, you may stop at any time. **All participants that complete the survey are invited to participate to a draw to win 50$.** Within two to four weeks after the completion of the survey, the prize will be drawn among participants. The research team will communicate with the winner by email or by phone. The prize will then be given by a member of the community.

Thank you for your interest!

Please read and sign the attached consent form.

The following questions will be used for the purpose of the draw. The answers will remain anonymous.

1. What is your name (for the draw)? ________________________________
2. How many dogs do you have?
   - ☐ 0
   - ☐ 1
   - ☐ 2
   - ☐ 3
   - ☐ 4
   - ☐ 5 or more

Dog owners section:

The following section includes specific questions on your dog(s). Please answer for each one of your dogs if you have more than one (for a maximum of four dogs). If you have more than four dogs, please focus on the ones you have had for the longest time.

Skip to <u>question 23</u> if you don't have dogs.

Questions	Dog 1	Dog 2	Dog 3	Dog 4
3. What is its name?				
4. Is it a male or a female?	☐ Male<br>☐ Female	☐ Male<br>☐ Female	☐ Male<br>☐ Female	☐ Male<br>☐ Female
5. What is its breed?				
6. Is it neutered (not able to have puppies)?	☐ Yes<br>☐ No<br>☐ Don't know	☐ Yes<br>☐ No<br>☐ Don't know	☐ Yes<br>☐ No<br>☐ Don't know	☐ Yes<br>☐ No<br>☐ Don't know
7. How old is it?				
8. What do you use this dog for? ***(Read options and check all that apply)***	☐ Guard dog<br>☐ Hunting<br>☐ Companion<br>☐ Sled<br>☐ Other:	☐ Guard dog<br>☐ Hunting<br>☐ Companion<br>☐ Sled<br>☐ Other:	☐ Guard dog<br>☐ Hunting<br>☐ Companion<br>☐ Sled<br>☐ Other:	☐ Guard dog<br>☐ Hunting<br>☐ Companion<br>☐ Sled<br>☐ Other:
9. Has it ever been vaccinated against rabies?	☐ Yes<br>☐ No<br>☐ Don't know	☐ Yes<br>☐ No<br>☐ Don't know	☐ Yes<br>☐ No<br>☐ Don't know	☐ Yes<br>☐ No<br>☐ Don't know
10. ***If yes***, was it within the last 12 months?	☐ Yes<br>☐ No<br>☐ Don't know	☐ Yes<br>☐ No<br>☐ Don't know	☐ Yes<br>☐ No<br>☐ Don't know	☐ Yes<br>☐ No<br>☐ Don't know

The following section includes general questions that may apply to all your dogs. If you have different answers for your dogs, please choose the answer that represents your older dog.

11. Where does(do) your dog(s) live?
    - ☐ Mostly indoors
    - ☐ Mostly outdoors
12. When your dog (s) is (are) outside, is it (are they) mainly?
    - ☐ Tied up (with a chain, a rope or a leash)
    - ☐ In a fenced area
    - ☐ Free-roaming
    - ☐ Other:______________
13. Do you let your dog roam free on occasion?
    - ☐ Yes
    - ☐ No

14. ***If yes:*** at what time do you let your dog wander freely?

	**During the week**	**During the week-end**	**When you are leaving the community**	**Never**
**In the morning**	☐	☐	☐	☐
**All day**	☐	☐	☐	☐
**In the evening**	☐	☐	☐	☐
**At night**	☐	☐	☐	☐

15. Has(have) your dog(s) ever been injured or sick?
    - ☐ Yes
    - ☐ No
    - ☐ Don't know
16. Has (have) your dog(s) ever been seen by a veterinarian?
    - ☐ Yes
    - ☐ No
    - ☐ Don't know
17. ***If yes***, was it by:
    - ☐ a veterinarian who visited your community
    - ☐ a veterinarian from outside your community
18. What dog services are available in your community?
    - ☐ Vaccination against rabies
    - ☐ Vaccination against other diseases
    - ☐ Sterilization (not able to have puppies)
    - ☐ Access to deworming medications
    - ☐ Urgent care
    - ☐ Euthanasia (ending life for pain relieving)
    - ☐ None of the above
    - ☐ Other : ________________
19. Do you find that the supply of veterinary services in your community is sufficient?
    - ☐ Yes
    - ☐ No
20. Is it important for you that your dog(s) get vaccinated?
    - ☐ Yes
    - ☐ No
    - ☐ Don't know/no opinion
21. Which dog services would you like to have available in your community?
    - ☐ Vaccination against rabies
    - ☐ Vaccination against other diseases
    - ☐ Sterilization (not able to have puppies)
    - ☐ Access to deworming medications
    - ☐ Urgent care
    - ☐ Euthanasia (ending life for pain relieving)
    - ☐ Dog refugee
    - ☐ Dog obedience training
    - ☐ Dog education for children or other
    - ☐ None of the above
    - ☐ Other : ________________
22. Would you use a veterinarian service that includes to get your intact dog not able to have puppies, if it was available in your community? ***(check all that apply)***
    - ☐ Yes, for my female(s)
    - ☐ Yes, for my male(s)
    - ☐ No

Dog owners and non-dog owners section:

The following questions are general question about interactions.

23. Have you, or any person of your entourage, ever been bitten by a dog ***(check all that apply***)?
    - ☐ No
    - ☐ Yes, me
    - ☐ Yes, at least one other adult of my entourage
    - ☐ Yes, at least one child of my entourage
24. ***If yes***, was it in the last 12 months? (If more than one option was selected for the previous question, **please consider the most recent bite**.)
    - ☐ Yes
    - ☐ No

The following questions are on your most recent bite. Skip to <u>question 30</u> if you have never been bitten.

25. Considering the most recent bite. Did you know the dog that bit you?
    - ☐ Yes, it was my dog.
    - ☐ Yes, it was a dog that belongs to someone from my surroundings.
    - ☐ No
    - ☐ Other: ________________
26. The dog that bit you was:
    - ☐ Free-roaming
    - ☐ Tied up
    - ☐ On a leash
    - ☐ Other: ________________
27. What did you do after you were bitten? I have:
    - ☐ Not done anything special
    - ☐ Rinsed /cleaned the wound
    - ☐ Disinfected the wound with a disinfectant (example: alcohol)
    - ☐ Put a bandage
    - ☐ Consulted a nurse or doctor
    - ☐ Consulted another member of my community
    - ☐ Killed the dog
    - ☐ Contacted a member of my community to kill the dog
    - ☐ Other : ______________
28. Did you consult a health professional (nurse, doctor) after this bite?
    - ☐ Yes
    - ☐ No
29. ***If yes***, did you get a vaccine after this bite?
    - ☐ Yes
    - ☐ No

The following questions are general question about perceptions.

30. Before completing this survey, have you ever heard about a disease called rabies?
    - ☐ Yes
    - ☐ No
31. How much do you know about rabies?
    - ☐ Never heard of rabies
    - ☐ Little knowledge
    - ☐ Basic knowledge
    - ☐ Extensive knowledge
32. Are you at risk of contracting rabies in your community?
    - ☐ Yes
    - ☐ No
    - ☐ Don’t know
33. You like dogs:
    - ☐ Strongly Disagree
    - ☐ Moderately Disagree
    - ☐ Unsure
    - ☐ Moderately Agree
    - ☐ Strongly Agree
34. Your dog is happy:
    - ☐ Strongly Disagree
    - ☐ Moderately Disagree
    - ☐ Unsure
    - ☐ Moderately Agree
    - ☐ Strongly Agree
35. Your dog is healthy:
    - ☐ Strongly Disagree
    - ☐ Moderately Disagree
    - ☐ Unsure
    - ☐ Moderately Agree
    - ☐ Strongly Agree
36. When a dog bites someone it should be killed:
    - ☐ Strongly Disagree
    - ☐ Moderately Disagree
    - ☐ Unsure
    - ☐ Moderately Agree
    - ☐ Strongly Agree
37. You are afraid of dogs:
    - ☐ Strongly Disagree
    - ☐ Moderately Disagree
    - ☐ Unsure
    - ☐ Moderately Agree
    - ☐ Strongly Agree

38. There are too many dogs in your community:
- ☐ Strongly Disagree
- ☐ Moderately Disagree
- ☐ Unsure
- ☐ Moderately Agree
- ☐ Strongly Agree

39. Dogs can transmit diseases:
- ☐ Strongly Disagree
- ☐ Moderately Disagree
- ☐ Unsure
- ☐ Moderately Agree
- ☐ Strongly Agree

40. Dog bites are a serious health problem:
- ☐ Strongly Disagree
- ☐ Moderately Disagree
- ☐ Unsure
- ☐ Moderately Agree
- ☐ Strongly Agree

41. The risk of being bitten by a dog in or around my community is high:
- ☐ Strongly Disagree
- ☐ Moderately Disagree
- ☐ Unsure
- ☐ Moderately Agree
- ☐ Strongly Agree

42. Dogs are important for people in my community:
- ☐ Strongly Disagree
- ☐ Moderately Disagree
- ☐ Unsure
- ☐ Moderately Agree
- ☐ Strongly Agree

43. Dogs in my community should be sterilized:
- ☐ Strongly Disagree
- ☐ Moderately Disagree
- ☐ Unsure
- ☐ Moderately Agree
- ☐ Strongly Agree

44. Rabies is a severe disease:
- ☐ Strongly Disagree
- ☐ Moderately Disagree
- ☐ Unsure
- ☐ Moderately Agree
- ☐ Strongly Agree

45. Having more veterinary services in my community will help us take care of our dogs:
- ☐ Strongly Disagree
- ☐ Moderately Disagree
- ☐ Unsure
- ☐ Moderately Agree
- ☐ Strongly Agree

46. The risk of contracting rabies in or around my community is high:
- ☐ Strongly Disagree
- ☐ Moderately Disagree
- ☐ Unsure
- ☐ Moderately Agree
- ☐ Strongly Agree

47. You are worried that you or one member of your family is at risk of contracting rabies:
- ☐ Strongly Disagree
- ☐ Moderately Disagree
- ☐ Unsure
- ☐ Moderately Agree
- ☐ Strongly Agree

48. It is easy for me to protect myself against contracting rabies:
- ☐ Strongly Disagree
- ☐ Moderately Disagree
- ☐ Unsure
- ☐ Moderately Agree
- ☐ Strongly Agree

49. It is easy for me to protect members of my family against contracting rabies:
- ☐ Strongly Disagree
- ☐ Moderately Disagree
- ☐ Unsure
- ☐ Moderately Agree
- ☐ Strongly Agree

Demography section:

50. Are you a beneficiary of the *James Bay and Nothern Quebec Agreement* (JBNQA)?
    - ☐ Yes
    - ☐ No
51. How long have you lived in (your municipality)? ________________
52. What is your main occupation?_________________
53. To which gender do you most identify:
    - ☐ A man
    - ☐ A woman
    - ☐ I prefer not to answer.
    - ☐ Not listed : ______________
54. What is your birth year? ________________
55. Do you have any comments or issues about dog health that you would like to mention?

    ____

    ____

    ____

56. Can we contact you by email or phone in the next days to conduct a 30 minutes interview on the same topics in order to better understand your needs and experiences? (It does not commit you in any way, another consent will be asked at the time of the interview.)
    - ☐ Yes, by email (enter your email address): ___________________________________
    - ☐ Yes, by phone (enter your phone number): ________________________
    - ☐ No

Thank you very much for your participation.

The overall results of this survey will be shared using poster and social media such as your community's Facebook page. The results will also be published in a scientific journal.

If you have any questions or comments, please write to Laurence Daigle on Facebook or write to one of the following address email:

laurence.daigle.1@umontreal.ca

cecile.aenishaenslin@umontreal.ca

ENGLISH VERSION

INTERVIEW QUESTIONS

Patients of the communities (beneficiaries et non-beneficiaries)

<u>**Introduction**</u>
Tell me how do you live with dogs around?
<u>**Experience with dogs bites**</u>
Tell me what happened when you were bitten?<br>What have you done? (Have you used health services in your community? If so, why and how did it happen? What happened to the dog?)
<u>**Perceptions of risks of contracting rabies**</u>
Have you ever heard of rabies? What do you know about rabies?<br>Have you thought about the risk of transmitting the rabies virus when you got bitten?<br>Currently, do you find that you, your community, are sufficiently informed about the risks associated with dog bites? (Risk of contracting rabies)
<u>**Perspectives for service improvement**</u>
How could we improve the services you received when you were bitten by a dog?<br>Are there other things that could be improved in your community to reduce the risk of bites and their consequences?

Residents of communities with a dog that has bitten a person (beneficiaries et non-beneficiaries)

<u>**Introduction**</u>
Tell me how do you live with dogs around?
<u>**Experience with dogs bites**</u>
Tell me what happened when your dog bit someone?<br>What happened next? (What did you do, what happened to the dog)
<u>**Perceptions of the dogs situation**</u>
Tell me about dogs here?<br>Tell me how do you live with dogs around? (situation of dogs in Nunavik; too much; free-roaming; afraid?)
<u>**Perceptions of risks of contracting rabies**</u>
Have you ever heard of rabies? What do you know about rabies?<br>Have you thought about the risk of transmitting the rabies virus when your dog bit someone?<br>Currently, do you find that you, your community, are sufficiently informed about the risks associated with dog bites? (Risk of contracting rabies)
<u>**Perspectives for service improvement**</u>
How could we improve the management of dogs during a bite event in your community?<br>Are there other things that could be improved in your community to reduce the risk of bites and their consequences?

Health care professionnals

<u>**Introduction**</u>
Can you briefly explain your role in managing dog bites in your community?
<u>**Role of health professionals, dog bites and zoonosis**</u>
Have you had any patients who have consulted for a situation related to dog bites?<br>What do you do / what would you do when a patient shows up with a dog bite? (do you think of the risk of transmission of the rabies virus when a patient presents with a dog bite)<br>When you arrived in the community, did you have all the information you would have liked to know about the patient bitten?
<u>**Perspectives for service improvement**</u>
What could be improved in your community to improve the management of dog bites and the associated risks?<br>Are there other things that could be improved in your community to reduce the risk of bites and their consequences?
