# Supplementary material for: Knowledge, attitudes, and practices regarding dogs and dog bites in Indigenous northern communities: A mixed methods study
Source: Front Vet Sci. 2023 Feb 20;10:1080152. doi: 10.3389/fvets.2023.1080152 (PMC9986472; doi:10.3389/fvets.2023.1080152)
Supplement: Supplementary file 2 [file Data_Sheet_2.PDF]

Supplementary Material – Appendices 2 to 7

Appendix 2. Veterinary services

Table 6. Veterinary services already present and those that dog owners would like to have.*

	KWW n (%)	MLJ-SCH n (%)	Total n (%)
**My dog has been sick/injured**	3/13 (23%)	16/29 (55%)	19/42 (45%)
**My dog has seen a vet**	9/14 (64%)	24/29 (83%)	33/43 (77%)
Who visited	7/9 (78%)	19/25 (76%)	26/34 (76%)
Outside	2/9 (22%)	7/25 (28%)	9/34 (26%)
**Services available in the community**			
Vaccination rabies	4/12 (33%)	9/29 (31%)	13/41 (32%)
Vaccination other	1/12 (8%)	6/29 (21%)	7/41 (17%)
Sterilization	3/12 (25%)	9/29 (31%)	12/41 (29%)
Deworming	2/12 (17%)	5/29 (17%)	7/41 (17%)
Urgent care	0	1/29 (3%)	1/41 (2%)
Euthanasia	0	1/29 (3%)	1/41 (2%)
None	7/12 (58%)	16/29 (55%)	23/41 (56%)
Other	1/12 (8%)	0	1/41 (2%)
**Veterinary services are sufficient**	2/13 (15%)	4/29 (14%)	6/42 (14%)
**It is important that my dog get vaccinated**	13/13 (100%)	28/29 (97%)	41/42 (98%)
**Services that I would like to have in my community**			
Vaccination rabies	9/13 (69%)	22/27 (82%)	31/40 (78%)
Vaccination other	12/13 (92%)	19/27 (70%)	31/40 (78%)
Sterilization	10/13 (77%)	18/27 (67%)	28/40 (70%)
Deworming	9/13 (69%)	19/27 (70%)	28/40 (70%)
Urgent care	10/13 (77%)	21/27 (78%)	31/40 (78%)
Euthanasia	6/13 (46%)	12/27 (44%)	18/40 (45%)
Dog refugee	5/13 (39%)	19/27 (70%)	24/40 (60%)
Dog obedience training	9/13 (69%)	14/27 (52%)	23/40 (58%)
Dog education program for children/others	9/13 (69%)	15/27 (56%)	24/40 (60%)
None	1/13 (8%)	0	1/40 (3%)
Other	1/13 (8%)	1/27 (4%)	2/40 (5%)
**If available, I would use sterilization/contraception**	10/11 (91%)	22/28 (79%)	32/39 (82%)
Yes, for my female	1/10 (10%)	4/22 (18%)	5/32 (16%)
Yes, for my male	4/10 (40%)	13/22 (59%)	17/32 (53%)
Yes, for both	5/10 (50%)	5/22 (23%)	10/32 (31%)

*No significant difference between the two localities.

Appendix 3. Model C – Exposure to dog bites

Table 7. Factors associated with (C) exposure to dog bites (n=104).

C) Determinants of exposure to dog bites

	**OR**	**95% CI**
Community (KWW: ref)	0.560	(0.212 – 1.483)
Gender (Woman: ref)	1.245	(0.448 – 3.457)
Age		
18-29 yr	1.217	(0.362 – 4.095)
30-39 yr	0.485	(0.086 – 2.720)
40-49 yr	3.274	(0.850 – 12.605)
50+ yr[R]	1	1

[R]Reference categories.

Appendix 4. Age and gender comparison between the sample and the census

Figure 4. Age distribution per gender from the sample data presented and compared to the demographic data from the statistic census, in Kawawachikamach*.

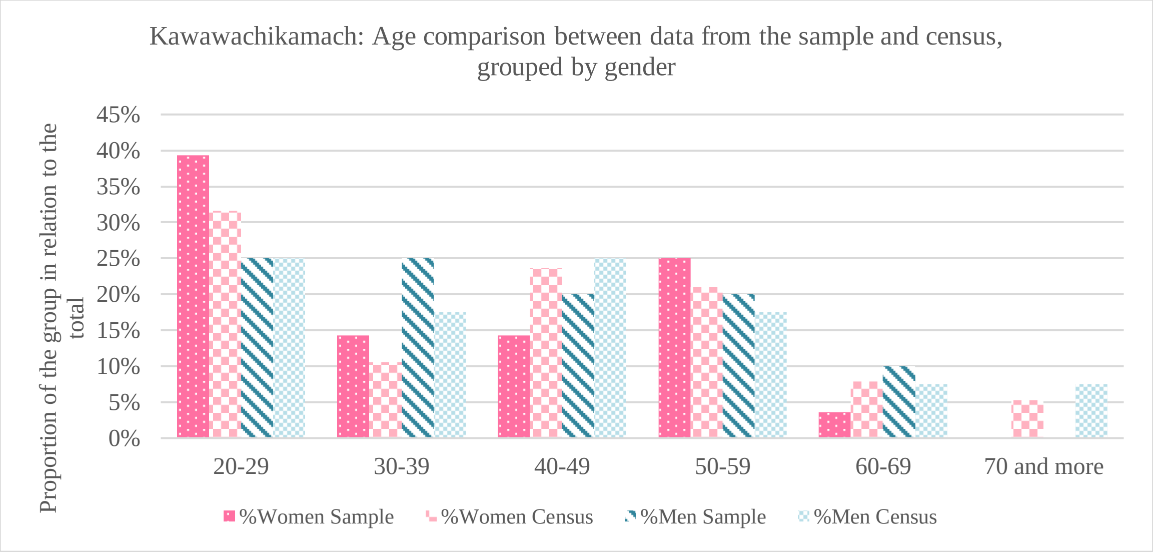

Figure 5. Age distribution per gender from the sample data presented and compared to the demographic data from the statistic census, in Matimekush-Lac John and Schefferville*.

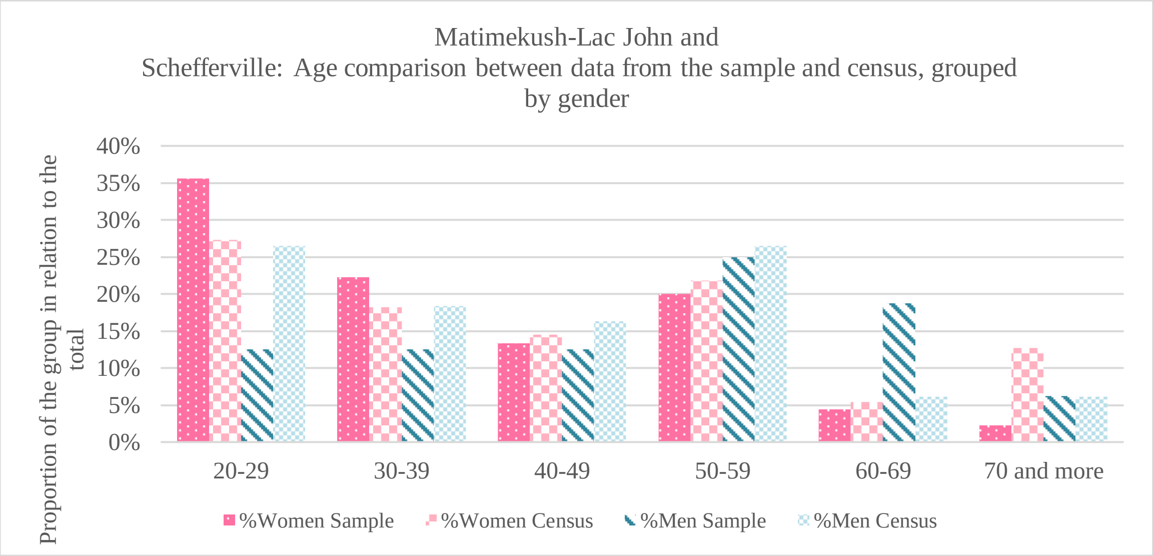

*Less than 20-year-old age category was removed from the graphics since census data between 18 and 20 years old only were not available.

Appendix 5. Results of perception variables

Perception on different questions are presented in Figure 6 and Figure 7. In surveys, in KWW and MLJ-SCH respectively, 28/53 (53 %) and 38/63 (60 %) had a little or no knowledge of rabies and 25/53 (47 %) and 25/63 (40 %) had a basic or extensive knowledge. There was no significant difference with the knowledge of rabies and either they judged to be at risk of contracting rabies or not between communities ($p > 0.05$). There is also no correlation between those two variables ($p > 0.05$).

The majority of survey owner respondents strongly agreed that their dog is happy and healthy and there was a significant difference between both localities; 7/16 (44 %) in KWW and 24/30 (80 %) in MLJ-SCH found their dog happy ($p < 0.05$) and 7/16 (44 %) in KWW and 23/30 (77 %) in MLJ-SCH found their dog healthy ($p < 0.05$).

Figure 6. Diverging Stacked Bar Chart of the Likert Scale related to Dog Perceptions, in Kawawachikamach.

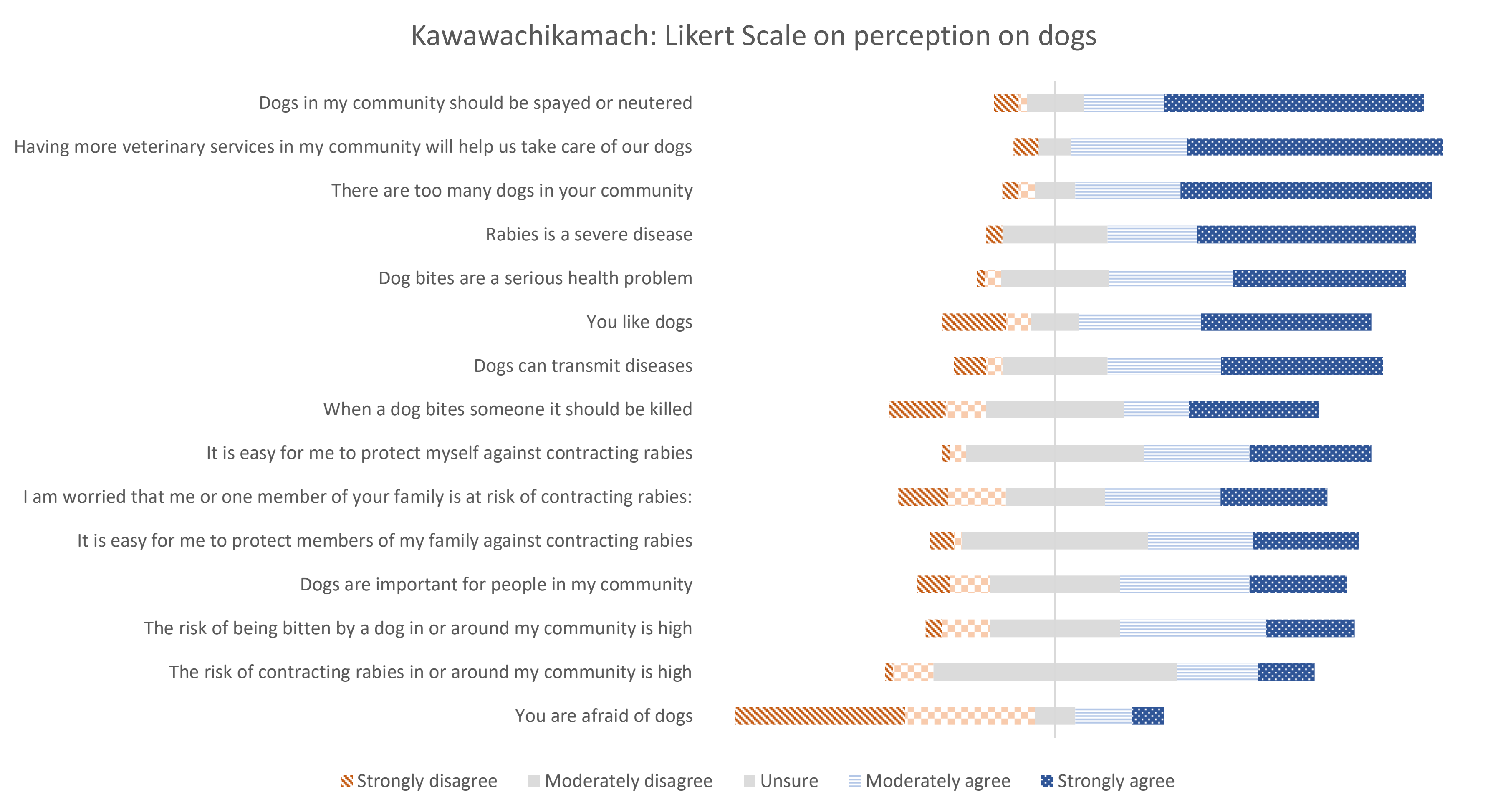

Figure 7. Diverging Stacked Bar Chart of the Likert Scale related to Dog Perceptions, in Matimekush-Lac John and Schefferville.

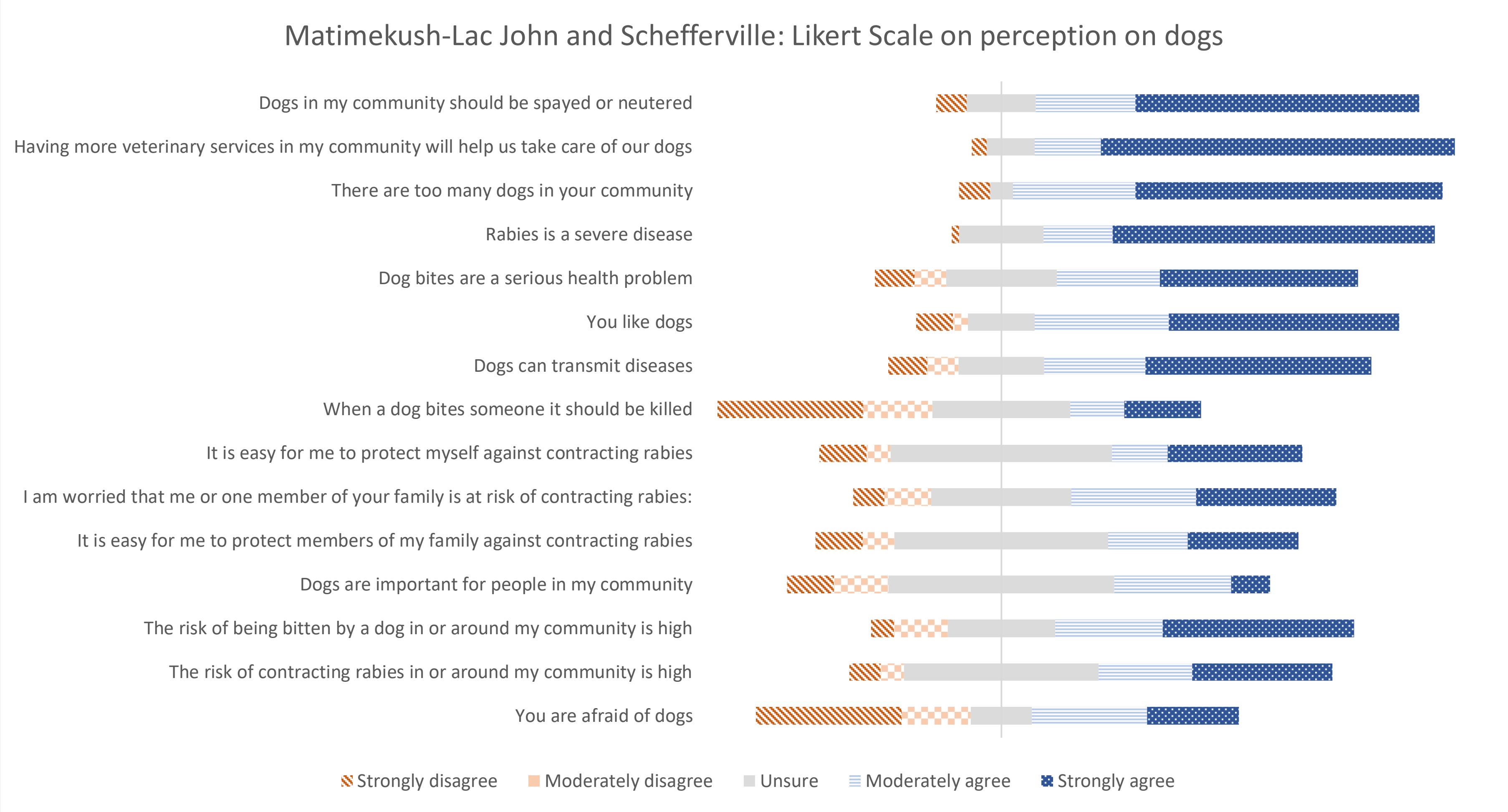

Appendix 6. Supplementary details on the exploratory factor analysis

The 15 perception variables presented in Figure 6 and Figure 7 were included in the initial EFA. All variables had initially a quality of representation (communality) superior to 0.2. Variable factor loadings were then examined in an iterative process, and variables with factor loadings inferior to 0.5 and/or with a lack of conceptual meaning in a group were excluded: “When a dog bites someone it should be killed”, “Dogs are important for people in my community”, “Having more veterinary services in my community will help us take care of our dogs”, “You like dogs”, “You are afraid of dogs”, “Rabies is a severe disease”, “Dogs in my community should be spayed or neutered”.

Table 8. Exploratory factor analysis of perception variables.

	K-MLJ-SCH	
**Initial model**		
**Quality of representation of EFA with fifteen variables (used for initial selection of variables)**		
You like dogs	0.315	
When a dog bites someone, it should be killed	0.253	
You are afraid of dogs	0.342	
There are too many dogs in your community	0.440	
Dogs can transmit diseases	0.535	
Dog bites are a serious health problem	0.373	
The risk of being bitten by a dog in, or around my community is high	0.452	
Dogs are important for people in my community	0.262	
Dogs in my community should be spayed or neutered	0.377	
Rabies is a severe disease	0.418	
Having more veterinary services in my community will help us take care of our dogs	0.208	
The risk of contracting rabies in or around my community is high	0.461	
I am worried that you or one member of your family are at risk of contracting rabies	0.406	
It is easy for me to protect myself against contracting rabies	0.558	
It is easy for me to protect members of my family against contracting rabies	0.566	
**Final model**		
Percentage of variance explained with the three latent factors	57.8 %	
	**Factor loadings**	**Correlation between variables and factors**
Dog risk perception (*F1*)		
There are too many dogs in your community	0.570	0.653
Dogs can transmit diseases	0.726	0.803
Dog bites are a serious health problem	0.598	0.661
The risk of being bitten by a dog in, or around my community is high	0.674	0.586

Perceived ability to protect oneself against rabies (*F2*)		
It is easy for me to protect myself against contracting rabies	0.577	0.824
It is easy for me to protect members of my family against contracting rabies	0.341	0.856
Rabies risk perception (*F3*)		
The risk of contracting rabies in or around my community is high	0.798	0.888
I am worried that you or one member of your family are at risk of contracting rabies	0.842	0.599
**Kaiser-Meyer-Olkin measure and Cronbach alpha**		
(*F1*) Kaiser-Meyer-Olkin measure of sampling adequacy	0.757	
(*F1*) Cronbach alpha	0.758	
(*F2*) Kaiser-Meyer-Olkin measure of sampling adequacy	0.500	
(*F2*) Cronbach alpha	0.836	
(*F3*) Kaiser-Meyer-Olkin measure of sampling adequacy	0.500	
(*F3*) Cronbach alpha	0.668	

Kaiser-Meyer-Olkin measure of sampling adequacy was 0.757 for Factor 1, 0.500 for Factor 2 and 0.500 for Factor 3.

Appendix 7. Description of the dog population owned

Table 9. Number of dogs owned (Cross-Sectional Survey).

	KWW<br>n (%)	MLJ-SCH<br>n (%)	Total<br>n (%)
**Total**	**56**	**66**	**122**
**Owner of one or more dogs**	**17/56 (30%)**	**30/66 (46%)**	**47/122 (39%)**
1 dog owned	14 (25%)	24 (36%)	38
2 dogs owned	0	2 (3%)	2
3 dogs owned	0	3 (5%)	3
4 dogs owned	1 (2%)	0	1
5 dogs owned	2 (4%)	1 (2%)	3

Table 10. Description of the Surveyed Dog Population (Cross-Sectional Survey).

	KWW<br>n (%)	MLJ-SCH<br>n (%)	Total<br>n (%)
**Sum of dogs reported by respondents**	**28**	**42**	**70**
**Number of dogs for which data were collected**$^{\alpha}$	**26**	**41**	**67**
**Sex**			
Male	11/14 (79%)	33/37 (89%)	44/51 (86%)
Female	3/14 (21%)	4/37 (11%)	7/51 (14%)
Missing data (unknown)	12	4	16
**Breed**			
Husky/husky mix	7/10 (70%)	13/22 (59%)	20/32 (63%)
Other	3/10 (30%)	9/22 (41%)	12/32 (38%)
Missing data (unknown)	16	19	35
**Neutered**	11/14 (79%)	32/36 (89%)	43/50 (93%)
Male	8/11 (73%)	28/32 (88%)	36/43 (84%)
Female	3/11 (27%)	4/32 (13%)	7/43 (16%)
Missing data (unknown)	12	6	18
**Age of dogs**			
<1 yo	3/11 (27%)	2/28 (7%)	5/39 (13%)
1-3 yo	5/11 (45%)	16/28 (57%)	21/39 (54%)
>3	3/11 (27%)	10/28 (36%)	13/39 (33%)
Missing data (unknown)	15	13	28
**Role**$^{\beta}$			
Guard	4/13 (31%)	14/35 (40%)	18/48 (38%)
Hunting	0/13	3/35 (9%)	3/48 (6%)
Companion	8/13 (62%)	31/35 (89%)*	39/48 (81%)

Sled	0/13	1/35 (3%)	1/48 (2%)
Other	4/13 (31%)	1/35 (3%)*	5/48 (10%)
Missing data (unknown)	13	6	19
**Vaccinated against rabies**	9/14 (64%)	30/37 (81%)	39/51 (76%)
Missing data (unknown)	14	8	22
In the last 12 months	6/9 (67%)	29/30 (97%)*	35/39 (90%)

$^{\alpha}$ One person could only answer for a maximum of four dogs (four oldest dogs that they owned).

$^{\beta}$ More than one answer was possible for this question.

* $p < 0.05$, ** $p < 0.01$, *** $p < 0.001$.
